# *N*′-(3,4-Dimethyl­benzyl­idene)furan-2-carbohydrazide

**DOI:** 10.1107/S1600536810027959

**Published:** 2010-07-17

**Authors:** Yu-Feng Li, Fang-Fang Jian

**Affiliations:** a Microscale Science Institute, Department of Chemistry and Chemical Engineering, Weifang University, Weifang 261061, People’s Republic of China; b Microscale Science Institute, Weifang University, Weifang 261061, People’s Republic of China

## Abstract

The title compound, C_14_H_14_N_2_O_2_, was prepared by the reaction of 3,4-dimethyl­benzaldehyde and furan-2-carbohydrazide. The dihedral angle between the aromatic rings is 35.48 (14)°. In the crystal, mol­ecules are linked by N—H⋯O hydrogen bonds, generating *C*(4) chains propagating in [010].

## Related literature

For a related structure, see: Li & Jian (2010 ▶).
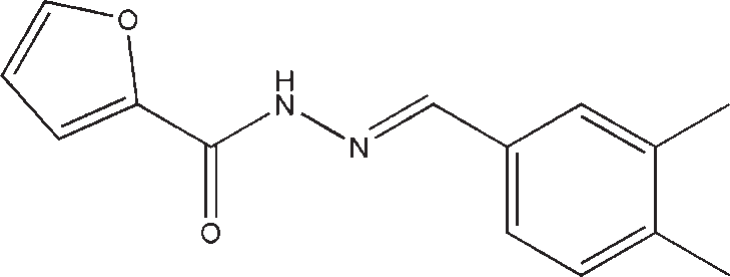


## Experimental

### 

#### Crystal data

C_14_H_14_N_2_O_2_
*M* *_r_* = 242.27
Orthorhombic, 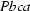

*a* = 17.655 (3) Å
*b* = 7.6304 (15) Å
*c* = 19.020 (4) Å
*V* = 2562.3 (9) Å^3^
*Z* = 8
Mo *K*α radiation
μ = 0.09 mm^−1^
*T* = 293 K
0.22 × 0.21 × 0.18 mm

#### Data collection

Bruker SMART CCD diffractometer
21744 measured reflections
2921 independent reflections
1563 reflections with *I* > 2σ(*I*)
*R*_int_ = 0.127

#### Refinement

*R*[*F* ^2^ > 2σ(*F* ^2^)] = 0.066
*wR*(*F* ^2^) = 0.177
*S* = 0.90
2921 reflections
163 parameters
H-atom parameters constrained
Δρ_max_ = 0.15 e Å^−3^
Δρ_min_ = −0.21 e Å^−3^

### 

Data collection: *SMART* (Bruker, 1997 ▶); cell refinement: *SAINT* (Bruker, 1997 ▶); data reduction: *SAINT*; program(s) used to solve structure: *SHELXS97* (Sheldrick, 2008 ▶); program(s) used to refine structure: *SHELXL97* (Sheldrick, 2008 ▶); molecular graphics: *SHELXTL* (Sheldrick, 2008 ▶); software used to prepare material for publication: *SHELXTL*.

## Supplementary Material

Crystal structure: contains datablocks global, I. DOI: 10.1107/S1600536810027959/hb5553sup1.cif

Structure factors: contains datablocks I. DOI: 10.1107/S1600536810027959/hb5553Isup2.hkl

Additional supplementary materials:  crystallographic information; 3D view; checkCIF report

## Figures and Tables

**Table 1:** Hydrogen-bond geometry (Å, °)

*D*—H⋯*A*	*D*—H	H⋯*A*	*D*⋯*A*	*D*—H⋯*A*
N2—H2*A*⋯O2^i^	0.86	2.06	2.921 (3)	174

Symmetry code: (i) 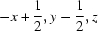.

**Fig. 1.**
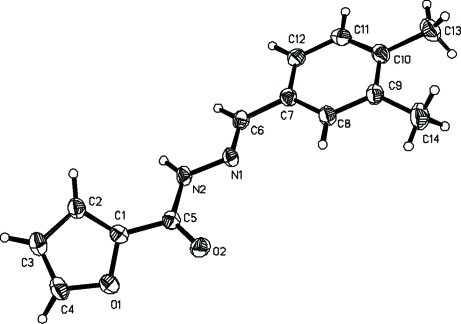
The molecular structure of (I) showing 30% probability displacement ellipsoids.

## supplementary crystallographic information

### Experimental

A mixture of 3,4-dimethylbenzaldehyde (0.1 mol), and furan-2-carbohydrazide (0.1 mol) was stirred in refluxing ethanol (20 mL) for 2 h to afford the title compound (0.090 mol, yield 90%). olourless blocks of (I) were obtained by recrystallization from ethanol at room temperature.

### Refinement

H atoms were fixed geometrically and allowed to ride on their attached atoms, with C—H distances=0.97 Å, and with *U*_iso_=1.2–1.5*U*_eq_.

### Crystal data

**Table:**

C_14_H_14_N_2_O_2_	*F*(000) = 1024
*M**_r_* = 242.27	*D*_x_ = 1.256 Mg m^−^^3^
Orthorhombic, *P**b**c**a*	Mo *K*α radiation, λ = 0.71073 Å
Hall symbol: -P 2ac 2ab	Cell parameters from 1563 reflections
*a* = 17.655 (3) Å	θ = 3.1–27.5°
*b* = 7.6304 (15) Å	µ = 0.09 mm^−^^1^
*c* = 19.020 (4) Å	*T* = 293 K
*V* = 2562.3 (9) Å^3^	Block, colorless
*Z* = 8	0.22 × 0.21 × 0.18 mm

### Data collection

**Table:**

Bruker SMART CCD diffractometer	1563 reflections with *I* > 2σ(*I*)
Radiation source: fine-focus sealed tube	*R*_int_ = 0.127
graphite	θ_max_ = 27.5°, θ_min_ = 3.1°
phi and ω scans	*h* = −22→22
21744 measured reflections	*k* = −9→9
2921 independent reflections	*l* = −24→23

### Refinement

**Table:**

Refinement on *F*^2^	Primary atom site location: structure-invariant direct methods
Least-squares matrix: full	Secondary atom site location: difference Fourier map
*R*[*F*^2^ > 2σ(*F*^2^)] = 0.066	Hydrogen site location: inferred from neighbouring sites
*wR*(*F*^2^) = 0.177	H-atom parameters constrained
*S* = 0.90	*w* = 1/[σ^2^(*F*_o_^2^) + (0.1*P*)^2^] where *P* = (*F*_o_^2^ + 2*F*_c_^2^)/3
2921 reflections	(Δ/σ)_max_ < 0.001
163 parameters	Δρ_max_ = 0.15 e Å^−^^3^
0 restraints	Δρ_min_ = −0.21 e Å^−^^3^

### Special details

**Table:**

Geometry. All e.s.d.'s (except the e.s.d. in the dihedral angle between two l.s. planes) are estimated using the full covariance matrix. The cell e.s.d.'s are taken into account individually in the estimation of e.s.d.'s in distances, angles and torsion angles; correlations between e.s.d.'s in cell parameters are only used when they are defined by crystal symmetry. An approximate (isotropic) treatment of cell e.s.d.'s is used for estimating e.s.d.'s involving l.s. planes.
Refinement. Refinement of *F*^2^ against ALL reflections. The weighted *R*-factor *wR* and goodness of fit *S* are based on *F*^2^, conventional *R*-factors *R* are based on *F*, with *F* set to zero for negative *F*^2^. The threshold expression of *F*^2^ > σ(*F*^2^) is used only for calculating *R*-factors(gt) *etc*. and is not relevant to the choice of reflections for refinement. *R*-factors based on *F*^2^ are statistically about twice as large as those based on *F*, and *R*- factors based on ALL data will be even larger.

### Fractional atomic coordinates and isotropic or equivalent isotropic displacement parameters (Å^2^)

**Table:**

	*x*	*y*	*z*	*U*_iso_*/*U*_eq_	
O2	0.34251 (10)	0.2908 (2)	0.40087 (10)	0.0493 (5)	
N2	0.23842 (12)	0.1209 (2)	0.38067 (10)	0.0404 (5)	
H2A	0.2147	0.0251	0.3897	0.048*	
N1	0.20989 (11)	0.2401 (3)	0.33290 (10)	0.0391 (5)	
C1	0.32632 (14)	0.0301 (3)	0.46664 (13)	0.0393 (6)	
C6	0.15127 (14)	0.1915 (3)	0.29934 (12)	0.0412 (6)	
H6A	0.1308	0.0813	0.3079	0.049*	
C5	0.30454 (14)	0.1586 (3)	0.41282 (12)	0.0384 (6)	
C7	0.11563 (14)	0.3062 (3)	0.24746 (12)	0.0397 (6)	
C9	0.11430 (15)	0.5784 (3)	0.18082 (13)	0.0456 (7)	
C4	0.40945 (19)	−0.1050 (4)	0.53228 (14)	0.0580 (8)	
H4A	0.4554	−0.1425	0.5508	0.070*	
C8	0.14661 (15)	0.4693 (3)	0.23064 (13)	0.0439 (7)	
H8A	0.1903	0.5058	0.2536	0.053*	
C2	0.28720 (16)	−0.0713 (3)	0.51175 (13)	0.0469 (7)	
H2B	0.2348	−0.0819	0.5147	0.056*	
C12	0.04994 (14)	0.2541 (3)	0.21381 (12)	0.0441 (7)	
H12A	0.0277	0.1472	0.2249	0.053*	
C11	0.01738 (15)	0.3619 (4)	0.16343 (13)	0.0475 (7)	
H11A	−0.0265	0.3252	0.1407	0.057*	
C3	0.34193 (18)	−0.1595 (4)	0.55411 (14)	0.0532 (7)	
H3A	0.3323	−0.2394	0.5899	0.064*	
C14	0.1495 (2)	0.7546 (4)	0.16590 (19)	0.0728 (10)	
H14A	0.1940	0.7691	0.1944	0.109*	
H14B	0.1138	0.8455	0.1767	0.109*	
H14C	0.1632	0.7614	0.1171	0.109*	
C13	0.01177 (19)	0.6353 (4)	0.09024 (15)	0.0709 (9)	
H13A	−0.0325	0.5771	0.0724	0.106*	
H13B	0.0470	0.6543	0.0525	0.106*	
H13C	−0.0025	0.7459	0.1103	0.106*	
O1	0.40208 (10)	0.0140 (3)	0.47873 (9)	0.0542 (6)	
C10	0.04847 (15)	0.5226 (4)	0.14611 (12)	0.0459 (7)	

### Atomic displacement parameters (Å^2^)

**Table:**

	*U*^11^	*U*^22^	*U*^33^	*U*^12^	*U*^13^	*U*^23^
O2	0.0437 (11)	0.0356 (10)	0.0687 (12)	−0.0029 (9)	0.0024 (8)	0.0115 (9)
N2	0.0499 (14)	0.0268 (11)	0.0444 (12)	−0.0013 (10)	−0.0043 (10)	0.0097 (9)
N1	0.0449 (13)	0.0310 (12)	0.0414 (12)	0.0036 (10)	−0.0041 (10)	0.0055 (9)
C1	0.0420 (15)	0.0323 (14)	0.0437 (14)	0.0024 (12)	−0.0047 (11)	−0.0028 (10)
C6	0.0483 (16)	0.0307 (14)	0.0446 (15)	−0.0021 (12)	0.0013 (12)	0.0039 (11)
C5	0.0420 (15)	0.0307 (14)	0.0424 (14)	0.0062 (12)	0.0032 (11)	−0.0005 (11)
C7	0.0424 (15)	0.0371 (15)	0.0395 (13)	0.0048 (11)	0.0014 (11)	0.0012 (11)
C9	0.0524 (17)	0.0404 (16)	0.0439 (14)	0.0035 (13)	0.0026 (12)	0.0060 (11)
C4	0.064 (2)	0.059 (2)	0.0503 (16)	0.0179 (17)	−0.0090 (14)	0.0095 (14)
C8	0.0466 (16)	0.0403 (16)	0.0448 (15)	0.0000 (13)	−0.0052 (12)	0.0042 (11)
C2	0.0533 (17)	0.0425 (16)	0.0450 (15)	−0.0056 (13)	−0.0088 (12)	0.0069 (12)
C12	0.0444 (16)	0.0435 (16)	0.0444 (15)	−0.0036 (13)	0.0012 (12)	−0.0010 (11)
C11	0.0379 (15)	0.0606 (19)	0.0440 (14)	0.0055 (14)	−0.0016 (11)	−0.0060 (13)
C3	0.068 (2)	0.0452 (17)	0.0461 (16)	0.0005 (15)	−0.0108 (14)	0.0060 (13)
C14	0.084 (2)	0.050 (2)	0.085 (2)	−0.0049 (18)	−0.0098 (18)	0.0256 (16)
C13	0.071 (2)	0.085 (2)	0.0564 (17)	0.0207 (19)	−0.0098 (16)	0.0175 (16)
O1	0.0446 (11)	0.0575 (13)	0.0604 (12)	0.0110 (10)	−0.0020 (9)	0.0143 (9)
C10	0.0486 (16)	0.0547 (18)	0.0345 (13)	0.0138 (14)	0.0026 (11)	0.0035 (12)

### Geometric parameters (Å, °)

**Table:**

O2—C5	1.232 (3)	C4—H4A	0.9300
N2—C5	1.349 (3)	C8—H8A	0.9300
N2—N1	1.381 (3)	C2—C3	1.427 (4)
N2—H2A	0.8600	C2—H2B	0.9300
N1—C6	1.271 (3)	C12—C11	1.388 (4)
C1—C2	1.346 (4)	C12—H12A	0.9300
C1—O1	1.363 (3)	C11—C10	1.383 (4)
C1—C5	1.469 (3)	C11—H11A	0.9300
C6—C7	1.462 (3)	C3—H3A	0.9300
C6—H6A	0.9300	C14—H14A	0.9600
C7—C12	1.383 (3)	C14—H14B	0.9600
C7—C8	1.396 (4)	C14—H14C	0.9600
C9—C8	1.384 (3)	C13—C10	1.513 (4)
C9—C10	1.403 (4)	C13—H13A	0.9600
C9—C14	1.508 (4)	C13—H13B	0.9600
C4—C3	1.329 (4)	C13—H13C	0.9600
C4—O1	1.371 (3)		
			
C5—N2—N1	118.3 (2)	C3—C2—H2B	126.8
C5—N2—H2A	120.9	C7—C12—C11	119.8 (2)
N1—N2—H2A	120.9	C7—C12—H12A	120.1
C6—N1—N2	115.8 (2)	C11—C12—H12A	120.1
C2—C1—O1	110.1 (2)	C10—C11—C12	121.7 (3)
C2—C1—C5	133.9 (2)	C10—C11—H11A	119.1
O1—C1—C5	115.8 (2)	C12—C11—H11A	119.1
N1—C6—C7	121.0 (2)	C4—C3—C2	106.5 (3)
N1—C6—H6A	119.5	C4—C3—H3A	126.8
C7—C6—H6A	119.5	C2—C3—H3A	126.8
O2—C5—N2	124.2 (2)	C9—C14—H14A	109.5
O2—C5—C1	122.2 (2)	C9—C14—H14B	109.5
N2—C5—C1	113.6 (2)	H14A—C14—H14B	109.5
C12—C7—C8	118.6 (2)	C9—C14—H14C	109.5
C12—C7—C6	120.1 (2)	H14A—C14—H14C	109.5
C8—C7—C6	121.3 (2)	H14B—C14—H14C	109.5
C8—C9—C10	118.7 (2)	C10—C13—H13A	109.5
C8—C9—C14	119.7 (3)	C10—C13—H13B	109.5
C10—C9—C14	121.6 (2)	H13A—C13—H13B	109.5
C3—C4—O1	110.8 (3)	C10—C13—H13C	109.5
C3—C4—H4A	124.6	H13A—C13—H13C	109.5
O1—C4—H4A	124.6	H13B—C13—H13C	109.5
C9—C8—C7	122.1 (2)	C1—O1—C4	106.2 (2)
C9—C8—H8A	118.9	C11—C10—C9	119.1 (2)
C7—C8—H8A	118.9	C11—C10—C13	120.1 (3)
C1—C2—C3	106.5 (3)	C9—C10—C13	120.9 (3)
C1—C2—H2B	126.8		

### Hydrogen-bond geometry (Å, °)

**Table:**

*D*—H···*A*	*D*—H	H···*A*	*D*···*A*	*D*—H···*A*
N2—H2A···O2^i^	0.86	2.06	2.921 (3)	174

Symmetry codes: (i) −*x*+1/2, *y*−1/2, *z*.
